# Effects of sigmoidoscopy screening (including colonoscopy) on colorectal cancer: A *meta*-analysis based on randomized controlled trials

**DOI:** 10.1016/j.pmedr.2024.102636

**Published:** 2024-02-01

**Authors:** Dongying Wang, Qian Xu, Senjie Dai, Yueming Zhang, Fulin Ding, Linling Ji

**Affiliations:** a The First Clinical Medical College, Zhejiang Chinese Medical University, Hangzhou, Zhejiang, China; b Department of Colorectal and Anal Surgery, The First Affiliated Hospital of Ningbo University, Ningbo, Zhejiang, China; c The Second Clinical Medical College, Zhejiang Chinese Medical University, Hangzhou, Zhejiang, China; d Intensive Care Unit, Hospital of Zhejiang People's Armed Police, Hangzhou, Zhejiang, China; e Outpatient Nursing, Ningbo Yinzhou No. 2 Hospital, Ningbo, Zhejiang, China

**Keywords:** Sigmoidoscopy, Colonoscopy, Colorectal Cancer, Incidence, Mortality, Meta-Analysis

## Abstract

**Background:**

This study aimed to investigate the role of endoscopy screening in colorectal cancer (CRC).

**Methods:**

Up to January 2023, databases were searched for studies related to sigmoidoscopy and colonoscopy screening. The incidence of CRC, and/or CRC mortality were the main observation outcomes.

**Results:**

A total of 5 randomized controlled trials (RCTs) published from 2017 to 2022 were included. Among them, four studies used sigmoidoscopy screening and one study involved colonoscopy screening. Statistical results showed that the incidence (RR: 0.78, p < 0.001) and mortality (RR: 0.75, p < 0.001) of CRC were significantly lower in the screening group than in the control group. Further, a subgroup analysis of CRC site indicated that the incidence and mortality of CRC in the screening group were significantly lower than those in the non-screened group, regardless of distal CRC (Incidence: RR: 0.66, p < 0.001; Mortality: RR: 0.62, p < 0.001) or proximal CRC (Incidence: RR: 0.94, p = 0.038; Mortality: RR: 0.89, p = 0.038). In terms of gender, compared with the non-screening group, both males (Incidence: RR: 0.73, p < 0.001; Mortality: RR: 0.68, p < 0.001) and females (Incidence: RR: 0.85, p < 0.001; Mortality: RR: 0.85, p = 0.017), the screening group had a significant decrease in the incidence and mortality of CRC.

**Conclusion:**

This *meta*-analysis demonstrated that sigmoidoscopy screening (including colonoscopy) could effectively reduce the incidence and mortality of CRC.

## 1 Introduction

Colorectal cancer (CRC) is the third most common malignant tumor and the second most lethal cancer (Xi and Xu, 2021). According to the GLOBOCAN database, the number of new cases and deaths of CRC accounted for 10 % of the total number of cancer cases, reaching 1.9 million and 935,000 respectively in 2020, posing an increasingly serious challenge to public health (Sung, 2021). Therefore, CRC screening is particularly a necessity. At present, the commonly used clinical screening approaches for CRC are fecal examination and colon structure examination. The former mainly includes fecal occult blood test, fecal genetics test, etc., while the latter mainly includes sigmoidoscopy, total colonoscopy barium enema, etc. (Navarro, 2017, Chan and Liang, 2022).

Falling within the field of endoscopy screening, sigmoidoscopy and total colonoscopy not only detect early cancers, but also effectively reduce CRC related mortality by early resection of adenomatous polyps and post-polypectomy surveillance (Rai and Mishra, 2018). More specifically, the sensitivity of sigmoidoscopy in CRC screening is 58 %-75 % (Anderson, 2004, Imperiale, 2000). The main reason for this sensitivity is the limitation of the probing scope, which leads to a controversial detection rate of proximal CRC (Atkin, 2010). However, sigmoidoscopy is more readily accepted by the general population because of its convenience, economy, and fewer postoperative adverse effects (Senore, 2011). Therefore, sigmoidoscopy is to some extent considered an alternative screening technique for colonoscopy. As far as colonoscopy is concerned, it is the gold standard for CRC screening. However, pain and discomfort lead to low compliance in the general population, which is a main obstacle (Bretthauer, 2016). Moreover, a cost-effectiveness analysis of a colon cancer screening strategy stated that the goal of large-scale colonoscopy screening could not be achieved due to insufficient investment and training (Barré, 2020). Thus, the benefits, adverse effects and cost-effectiveness of balanced endoscopy in CRC screening are of particular interest.

The international randomized controlled trials (RCTs) on endoscopic-based CRC screening are very limited. Previously published *meta*-analyses of sigmoidoscopy screening were mainly focused on the years 2009–2015, with a lack of timeliness of these studies (Swartz et al., 2019, Elmunzer, 2012, Shroff, 2014, Brenner et al., 2014, Holme, 2017). Additionally, a recent study by Zhang *et al*. had good consistency with previous studies regarding the reduction of CRC morbidity and mortality through sigmoidoscopy screening, yet some differences in subgroup analyses appear to remain (Zhang, 2023). For example, Zhang's study showed that sigmoidoscopy screening did reduce the incidence of CRC in males, unlike the study by Shroff *et al*. For proximal CRC, previous studies have also been controversial. Therefore, this study aimed to review previously published RCTs on sigmoidoscopy and/or colonoscopy screening in an attempt to explore evidence with higher quality for endoscopy screening in terms of CRC morbidity and mortality.

## 2 Methods

### 2.1 Search strategy

Guided by the Preferred Reporting Items for Systematic Reviews and Meta-Analyses (PRISMA), this *meta*-analysis was registered with the International prospective register of systematic reviews (PROSPERO) under the registration number CRD42023413007. This study was a *meta*-analysis and ethics approval seemed not applicable. As of January 2023, searches were conducted in five electronic databases, PubMed, EBSCO, Cochrane Library, Web of Science, and Embase, to explore the impact of sigmoidoscopy and colonoscopy screening on the morbidity as well as mortality of CRC, and/or all-cause mortality in the general population. Meanwhile, to avoid potential omissions, the references included in the study were further retrieved manually. The subject words and free words were as follows: (Sigmoidoscopy OR Colonoscopy) AND (Intestinal Neoplasm OR Neoplasm, Intestinal OR Neoplasms, Intestinal OR Intestines Neoplasms OR Intestines Neoplasm OR Neoplasm, Intestines OR Neoplasms, Intestines OR Cancer of Intestines OR Intestines Cancers OR Cancer of the Intestines OR Intestines Cancer OR Cancer, Intestines OR Cancers, Intestines OR Intestinal Cancer OR Cancer, Intestinal OR Cancers, Intestinal OR Intestinal Cancers OR Colorectal Neoplasm OR Neoplasm, Colorectal OR Neoplasms, Colorectal OR Colorectal Tumors OR Colorectal Tumor OR Tumor, Colorectal OR Tumors, Colorectal OR Colorectal Cancer OR Cancer, Colorectal OR Cancers, Colorectal OR Colorectal Cancers OR Colorectal Carcinoma OR Carcinoma, Colorectal OR Carcinomas, Colorectal OR Colorectal Carcinomas) and (Randomized Controlled Trial OR Random OR RCT).

### 2.2 Selection criteria

#### 2.2.1 Inclusion criteria

(1) General population with no history of CRC; (2) Participants who were involved in sigmoidoscopy and/or colonoscopy screening tests constituted the intervention group (including combined screening with fecal occult blood test), with the control group that did not receive endoscopy screening (but which included access to routine care); (3) The outcome indicators were: the incidence of CRC and/or CRC mortality; (4) The study type was RCT, and the relevant trial registration numbers were provided.

#### 2.2.2 Exclusion criteria

(1) The intervention group for other screening methods (such as fecal occult blood test, colon CT imaging technology); (2) Non-English articles; (3) Unavailability of full text; (4) Unavailability of data; (5) Updated studies (priority was given to including the most recently published study and/or the largest sample size study).

### 2.3 Data extraction

The requirements of the inclusion were strictly enforced, with two researchers independently screening the studies and summarizing relevant data. In case of disputes, proofreading was required and agreement was reached after discussion. For each study, information included was: study authors, year of publication, country of study, region of participant recruitment, time of participant recruitment, screening type of intervention, sample size, basic characteristics of participants, main outcomes and/or secondary outcomes, etc.

### 2.4 Quality assessment

Two researchers independently completed the quality assessment of the included studies. Since this study was based on RCTs, the Cochrane bias risk assessment tool was used (Higgins, 2011).

### 2.5 Outcome measures

This study examined the effectiveness of sigmoidoscopy and colonoscopy screening in the general population. Endoscopic screening procedures include but are not limited to flexible sigmoidoscopy, rigid sigmoidoscopy, and total colonoscopy. CRC is categorized as distal CRC or proximal CRC, with the incidence of CRC and/or CRC mortality as the primary outcomes, and all-cause mortality as a secondary outcome.

### 2.6 Statistical analysis

The pooled relative risk (RR) and 95 % confidence interval (95 %CI) were used to assess the effects of sigmoidoscopy and colonoscopy screening. Heterogeneity analysis was performed using the I^2^ test, with I^2^ ≤ 25 % as no heterogeneity, I^2^ between 25 % and 50 % as mild heterogeneity, I^2^ > 50 % and ≤ 75 % as moderate heterogeneity, and I^2^ > 75 % as severe heterogeneity (Czumbel, 2020). To improve the credibility of this *meta*-analysis, a random effect model was applied due to the different characteristics of the included studies (Cai and Fan, 2020). Additionally, subgroup analyses were performed to explore sources of heterogeneity based on the site involved in CRC (distal and proximal), the gender of the participants and the different sites of CRC across genders. Egger's test was applied to detect publication bias (Bowden et al., 2015). Simultaneously, sensitivity analysis was used to evaluate the stability of the results (Gu, 2019). All the above statistical results were processed using Stata12.0, requiring bilateral p values of less than 0.05 for statistical differences.

## 3 Results

### 3.1 Description of trials included

**Fig. 1:**
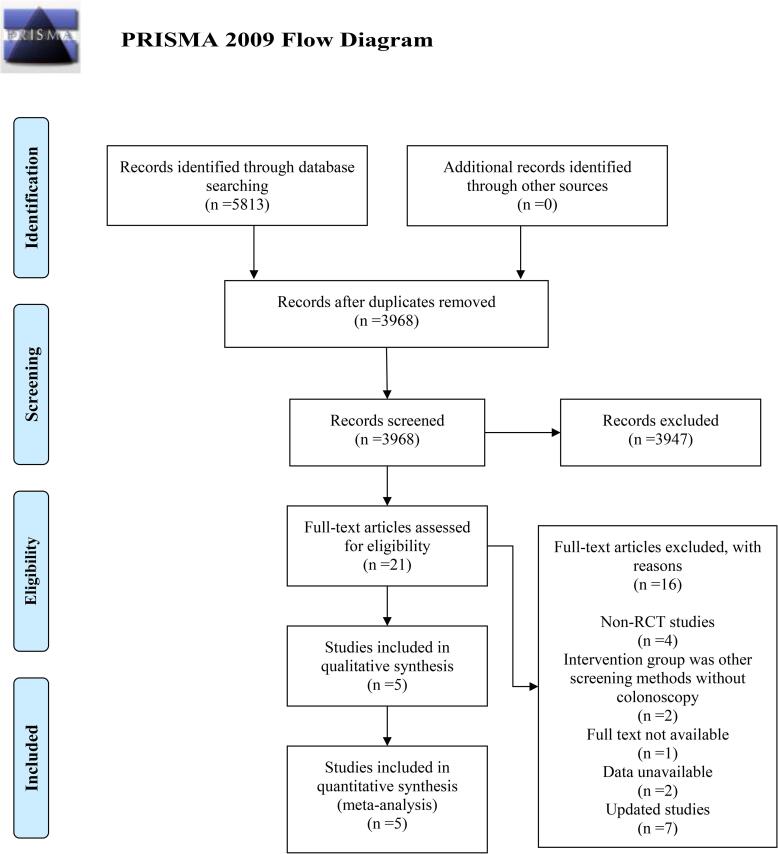
Flow diagram of selection.

According to the above retrieval formula, 5,813 search terms were obtained from 5 electronic databases. After eliminating duplicate studies, 3,968 studies were retained. Further reading the titles and abstracts, 3,947 studies unrelated to this study were removed. Among the remaining 21 studies, 4 non-RCTs were excluded, and 2 studies were excluded because other screening methods were implemented. 1 and 2 studies were excluded due to unavailability of full text or data, respectively. 7 studies were excluded owing to the priority for studies with the largest sample size and/or the most recent research. Finally, 5 RCTs met the inclusion criteria and were analyzed (Fig. 1) (Miller, 2019, Senore, 2022, Atkin, 2017, Holme, 2018, Bretthauer, 2022).

### 3.2 Characteristics of patients and trials

Five RCTs (four sigmoidoscopy studies and one colonoscopy study) published from 2017 to 2022 involved nearly 550,000 participants aged 50–74 years. Final sigmoidoscopy screening rates ranged from a high of 71.0 % to a low of 19.5 % in the intervention group. All five studies had a follow-up time greater than or equal to 10 years. In terms of distribution, the five studies were published in four countries, including two from Norway and one each in the United Kingdom, the United States and Italy. It covered participants from Britain, Poland, Norway, Sweden, the United States and Italy. The basic characteristics of the included studies were showed in Table 1 and Supplementary Table 1 in detail.

**Table 1:** **Characteristics of included studies.** NR: Not reported; FOBT: Fecal occult blood test; I: Intervention; C: Control.

Autor	Year	Country	Participant recruitment area	Recruitment time	Study	Intervention	No. of participants		Screening rate in intervention group	Follow-up time (Years, Median)	
							Intervention	Control		Incidence	Mortality
Atkin	2017	United Kingdom	14 UK hospitals	1994–1999	UKFSST	Flexible Sigmoidoscopy	57,098	112,936	71 %	17.1	
Bretthauer	2022	Norway	Poland; Norway; Sweden; Netherlands^a^	2009–2014	NordICC	Colonoscopy	28,220	56,365	42 %	10	
Holme	2018	Norway	Oslo and Telemark county	1999–2001	NORCCAP	Sigmoidoscopy; Sigmoidoscopy + FOBT	20,552	78,126	19.5 %	14.8	
Miller	2019	United States	10 clinical centers across the US	1993–2001	PLCO	Flexible Sigmoidoscopy	77,443	77,444	NR	I:15.9	I:16.9
										C:15.7	C:16.7
Senore	2022	Italy	6 centers in Italy^b^	1995–1999	SCORE	Flexible Sigmoidoscopy	17,136	17,136	57.8 %^c^	15.4	18.8

a 9780 participants, from the Netherlands, could not be included because Statistics Netherlands could not provide follow-up data from the usual-care group owing to a new Dutch law based on the recently introduced European Union General Data Protection Regulation;

b Turin, Biella, Genoa, Milan, Rimini, and Arezzo;

c 9911 participants in the intervention group had flexible sigmoidoscopy screening.

### 3.3 Quality of study and risk of bias

Of the five studies included, two studies did not explicitly state the use of blinding for subjects and testers, and were therefore identified to be 'unclear'. Besides, five studies showed 'low risk' in the remaining six aspects (Supplementary Fig. 1 and Supplementary Fig. 2).

**Fig. 2:**
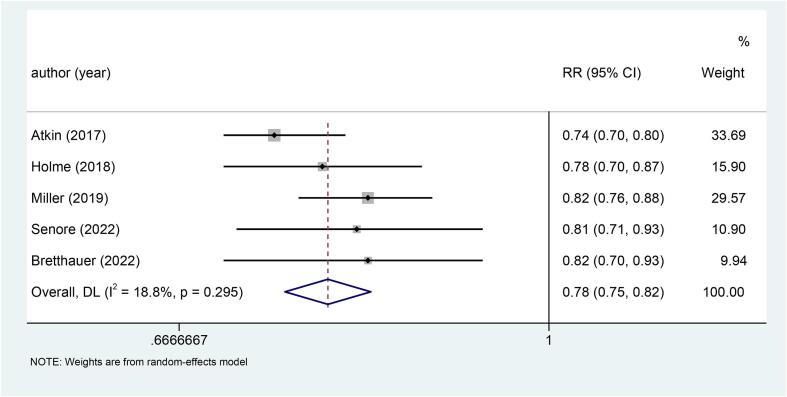
Forest plot of the relationship between sigmoidoscopy screening (including colonoscopy) and the incidence of CRC (p < 0.001).

### 3.4 Incidence and mortality of CRC

**Fig. 3:**
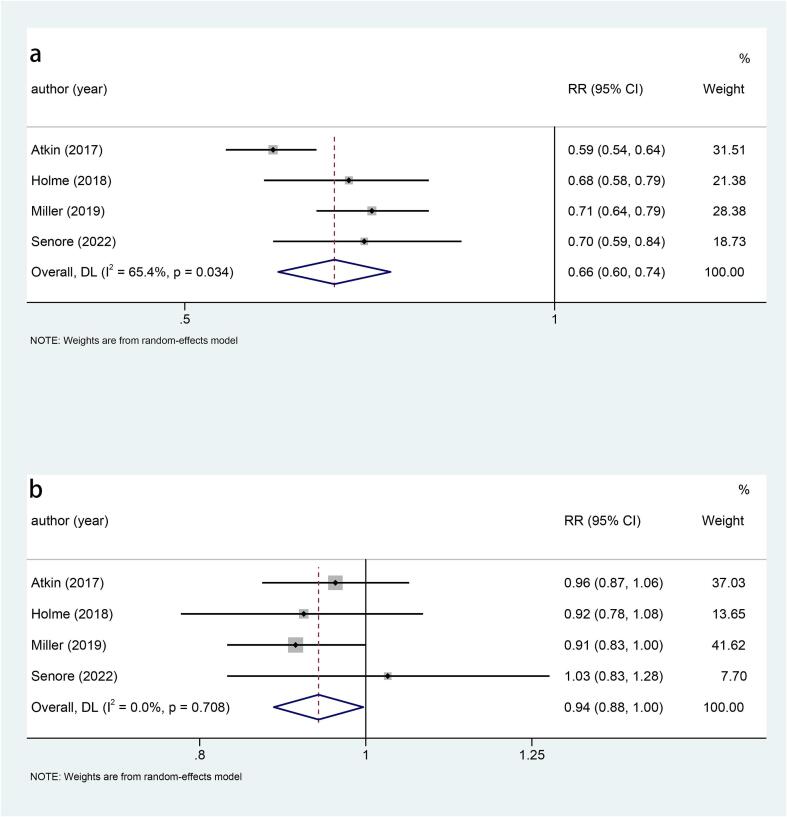
a, Forest plot of the relationship between sigmoidoscopy screening (including colonoscopy) and the incidence of distal CRC (p < 0.001); b, Forest plot of the relationship between sigmoidoscopy screening (including colonoscopy) and the incidence of proximal CRC (p = 0.038).

**Fig. 4:**
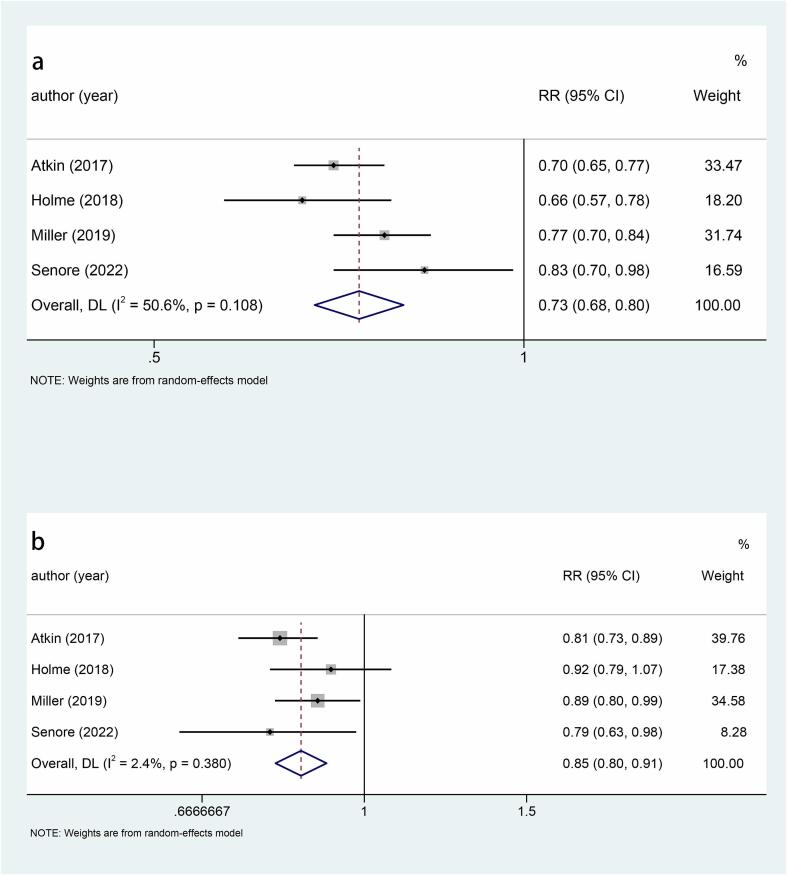
a, Forest plot of the relationship between sigmoidoscopy screening (including colonoscopy) and males CRC incidence (p < 0.001); b, Forest plot of the relationship between sigmoidoscopy screening (including colonoscopy) and females CRC incidence (p < 0.001).

Five studies explored the relationship between sigmoidoscopy screening (including colonoscopy) and the incidence of CRC. The results showed that the incidence of CRC was significantly lower in the screening group than in the control group (RR: 0.78, 95 %CI: 0.75–0.82, p < 0.001) (Fig. 2). Subgroup analysis of CRC site demonstrated a reduced incidence of distal CRC (RR: 0.66, 95 %CI: 0.60–0.74, p < 0.001) (Fig. 3a) and proximal CRC (RR: 0.94, 95 %CI: 0.88–1.00, p = 0.038) (Fig. 3b) in participants who underwent screening, compared to non-screened participants. Moreover, the decrease in distal CRC incidence was greater than that in proximal CRC incidence in screened participants. As for gender, this study indicated that the incidence was lower in both males (RR: 0.73, 95 %CI: 0.68–0.80, p < 0.001) (Fig. 4a) and females (RR: 0.85, 95 %CI: 0.80–0.91, p < 0.001) (Fig. 4b) in the intervention group compared to the control group, with males experiencing a greater decrease in CRC incidence than females. Combined with the above subgroup analyses, the intervention group showed a significant reduction in distal CRC in males (RR: 0.61, p < 0.001), distal CRC in females (RR: 0.73, p = 0.007), proximal CRC in males (RR: 0.88, p = 0.006) compared with the control group, while for females with proximal CRC (RR: 0.99, p = 0.832), there was no difference between the intervention group and the control group (Table 2).

**Table 2:** **Subgroup analysis of the relationship between the incidence of CRC and site/gender.** RR: Risk ratio; CI: Confidence interval; CRC: Colorectal cancer.

	No. of studies	RR	95 %CI	*p*	Heterogeneity (I^2^)
Male with distal CRC	3	0.61	0.56, 0.66	<0.001	0.0 %
Female with distal CRC	3	0.73	0.59, 0.92	0.007	77.3 %
Male with proximal CRC	3	0.88	0.81, 0.96	0.006	0.0 %
Female with proximal CRC	3	0.99	0.91, 1.08	0.832	0.0 %

**Fig. 5:**
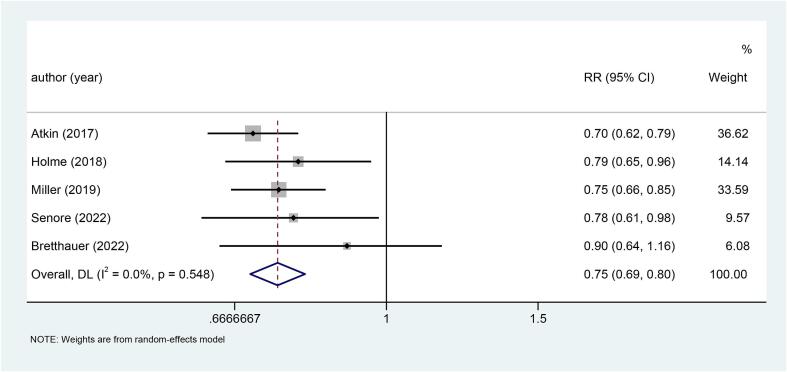
Forest plot of the relationship between sigmoidoscopy screening (including colonoscopy) and CRC mortality (p < 0.001).

**Fig. 6:**
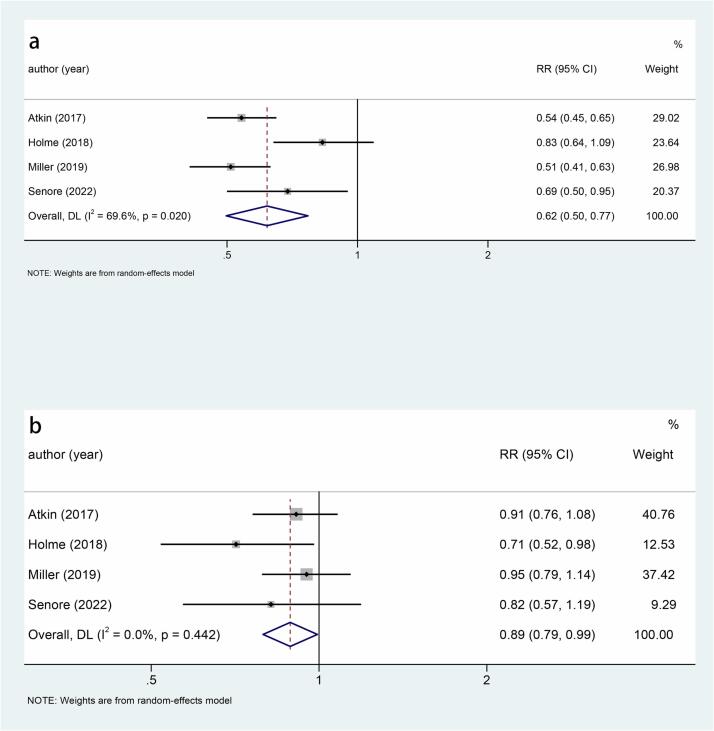
a, Forest plot of the relationship between sigmoidoscopy screening (including colonoscopy) and distal CRC mortality (p < 0.001); b, Forest plot of the relationship between sigmoidoscopy screening (including colonoscopy) and proximal CRC mortality (p = 0.038).

**Fig. 7:**
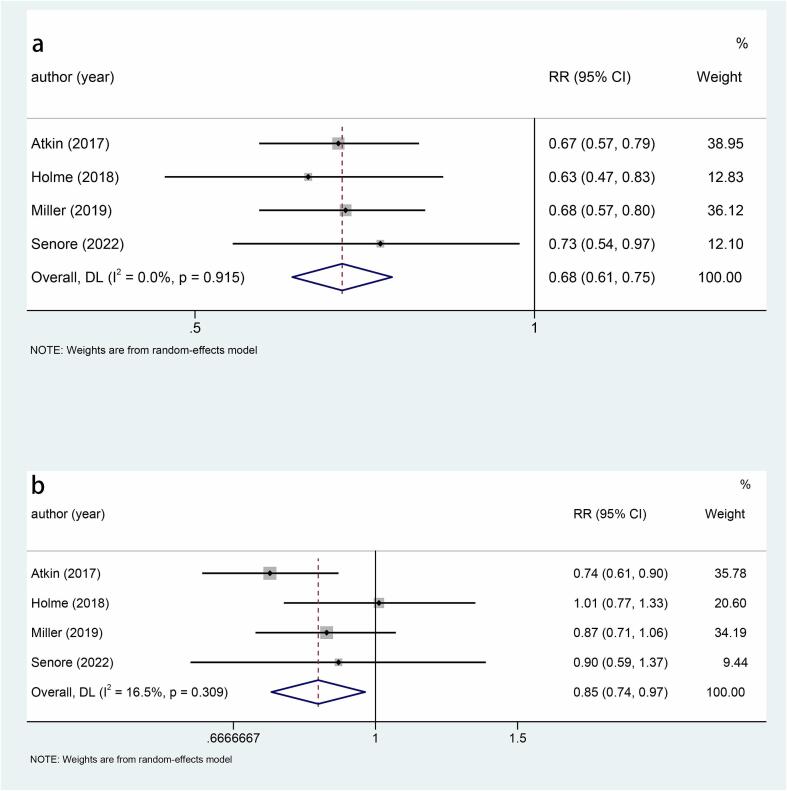
a, Forest plot of the relationship between sigmoidoscopy screening (including colonoscopy) and males CRC mortality (p < 0.001); b, Forest plot of the relationship between sigmoidoscopy screening (including colonoscopy) and females CRC mortality (p = 0.017).

For CRC mortality, five studies showed that participants who underwent sigmoidoscopy screening (including colonoscopy) had a significantly lower mortality rate than the control group (RR: 0.75, 95 %CI: 0.69–0.80, p < 0.001) (Fig. 5). In-depth subgroup analysis indicated that the intervention group significantly reduced the mortality rate of distal CRC (RR: 0.62, 95 %CI: 0.50–0.77, p < 0.001) (Fig. 6a) and proximal CRC (RR: 0.89, 95 %CI: 0.79–0.99, p = 0.038) (Fig. 6b), compared with the control group. Meanwhile, the range of reduction in mortality from distal CRC was more pronounced in the intervention group than proximal CRC. As for the relationship between gender and CRC mortality, statistical analysis found that both males (RR: 0.68, 95 %CI: 0.61–0.75, p < 0.001) (Fig. 7a) and females (RR: 0.85, 95 %CI: 0.74–0.97, p = 0.017) (Fig. 7b) were significantly lower in the intervention group than in the control group. After intervention, there was a greater reduction in CRC mortality in males than in females. Subgroup analysis was further performed by combining site and gender. Three studies illustrated that the mortality rate of distal CRC in males was significantly lower in the intervention group than in the control group (RR: 0.52, p < 0.001). However, there was no statistical difference between the two groups in females with distal CRC (RR: 0.73, p = 0.146) or proximal CRC (RR: 0.92, p = 0.322), or in males with proximal CRC (RR: 0.86, p = 0.150) (Table 3).

**Table 3:** **Subgroup analysis of the relationship between CRC mortality and site/gender.** RR: Risk ratio; CI: Confidence interval; CRC: Colorectal cancer.

	No. of studies	RR	95 %CI	*p*	Heterogeneity(I^2^)
Male with distal CRC	3	0.52	0.45, 0.61	<0.001	0.0 %
Female with distal CRC	3	0.73	0.48, 1.12	0.146	77.4 %
Male with proximal CRC	3	0.86	0.70, 1.06	0.150	30.5 %
Female with proximal CRC	3	0.92	0.78, 1.09	0.322	0.0 %

### 3.5 All-cause mortality

**Fig. 8:**
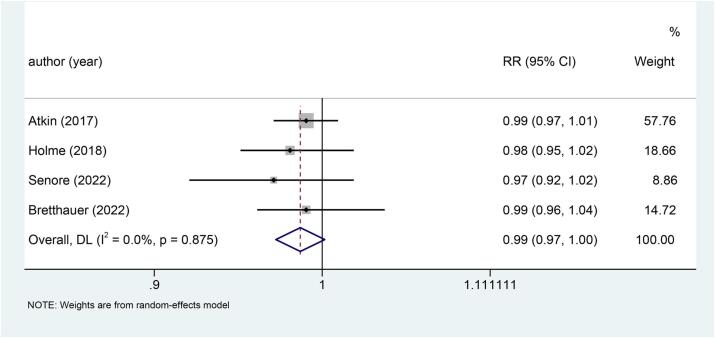
Forest plot of the relationship between sigmoidoscopy screening (including colonoscopy) and all-cause mortality (p = 0.079).

**Fig. 9:**
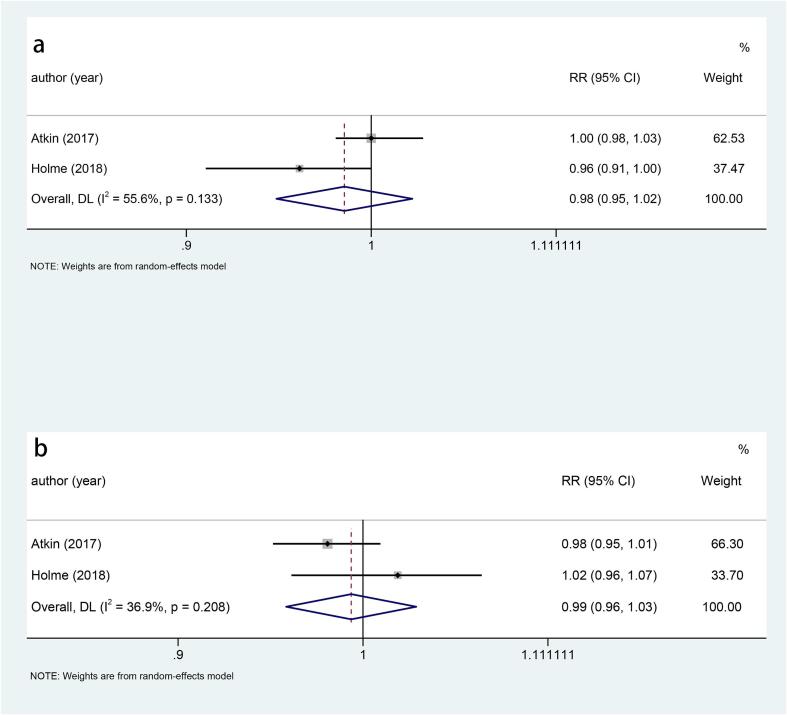
a, Forest plot of the relationship between sigmoidoscopy screening (including colonoscopy) and all-cause mortality in males (p = 0.439); b, Forest plot of the relationship between sigmoidoscopy screening (including colonoscopy) and all-cause mortality in females (p = 0.722).

Four studies examined the relationship between sigmoidoscopy screening (including colonoscopy) and all-cause mortality in the general population. The results indicated that there was no significant difference in all-cause mortality between the two groups (RR: 0.99, p = 0.079) (Fig. 8). Gender subgroup analysis found that there was no significant difference in all-cause mortality between the two groups, either males (RR: 0.98, p = 0.439) (Fig. 9a) or females (RR: 0.99, p = 0.722) (Fig. 9b).

### 3.6 Sensitivity analysis and publication bias

The stability of the results was evaluated using sensitivity analysis. After removing each study one by one, the statistical results on sigmoidoscopy screening (including colonoscopy) and CRC incidence were stable (Supplementary Fig. 3). Egger's test was used in this study to assess publication bias. There was no significant bias between studies involving sigmoidoscopy screening and the incidence of CRC (p = 0.435) (Supplementary Fig. 4). In terms of CRC mortality, sensitivity analysis showed that the results were stable (Supplementary Fig. 5). No publication bias was found (p = 0.051) (Supplementary Fig. 6). Sensitivity analysis also declared the statistical results between sigmoidoscopy screening and all-cause mortality to be stable (Supplementary Fig. 7). As for Egger's test, it showed no publication bias between these relevant studies (p = 0.228) (Supplementary Fig. 8).

## 4 Discussion

This *meta*-analysis examined five RCTs involving nearly 550,000 participants to explore the value of sigmoidoscopy screening (including colonoscopy) for CRC. The statistical results showed that sigmoidoscopy screening was effective in reducing the incidence and mortality of CRC in the general population, especially in distal CRC and in males. However, in terms of all-cause mortality, no role for sigmoidoscopy screening was found in this study.

This study found a 22 % reduction in the incidence of CRC and a 25 % reduction in mortality after sigmoidoscopy screening compared to controls. Sigmoidoscopy screening (including colonoscopy) can be a key reason for the timely detection and effective removal of precancerous lesions. It is well known that most CRCs are developed from polyps (Grady and Markowitz, 2015). As a result of the accumulation of genetic and epigenetic changes, tumor suppressor genes in stem cells are inactivated and oncogenes are activated, culminating in the formation of cancer stem cells, which are necessary for the formation and maintenance of tumors (Vogelstein, 1988). At present, colorectal precancerous lesions mainly involve two distinct pathways: chromosomal instability and microsatellite instability (Grady and Carethers, 2008). The former is present in 85 % of CRC, whereas the latter accounts for approximately 15 % of CRC and is usually found in serrated polyps (Grady and Markowitz, 2015). Shaukat *et al*. found that by performing diagnostic colonoscopy and polypectomy in those with a positive fecal occult blood test result, the probability of the participants being diagnosed with CRC decreased by about two-thirds over a 24-year follow-up period (Shaukat, 2021). It is speculated that further reductions in CRC mortality will be achieved through effective implementation of secondary prevention. A study by Corley *et al*. noted that the adenoma screening rate obtained by colonoscopy screening was negatively correlated with the mortality of CRC. For every 1 % increase in adenoma detection rate, the risk of fatal interval CRC decreased by 5 % (Corley, 2014). Thus, the significance of sigmoidoscopy screening (including colonoscopy) in reducing the incidence and mortality of CRC has been reaffirmed.

In terms of the site of CRC in this study, compared with the control group, sigmoidoscopy screening (including colonoscopy) reduced the incidence of distal CRC and proximal CRC by 34 % and 6 %, respectively, and the mortality rate decreased by 38 % and 11 %, respectively. On the one hand, since most of the screening in this study was carried out under sigmoidoscopy, which might be of little value for proximal CRC. It revealed a greater advantage in reducing the incidence and mortality of distal CRC compared to proximal CRC (Bretthauer, 2022). On the other hand, the differences in anatomy, histology and cancer morphology between distal and proximal CRC may also contribute. Abnormal crypt lesions, a hallmark of hyperplastic and adenomatous polyps, have been noted to be heterogeneous in distribution density as well as morphology across the proximal colon, distal colon and rectum (Roncucci et al., 1991). These are mainly manifested by denser distribution in the rectum and larger morphology in the distal colon (Roncucci, 1998, Arai and Kino, 1989). As a result, the precancerous lesions of the distal colon and rectum are more likely to be detected and removed at an early stage, reducing the incidence and mortality of distal CRC significantly. Additionally, Nawa *et al* observed that the early-stage cancers in the left colon (distal colon) were mainly polypoid, whereas early-stage cancers in the right colon (proximal colon) were mainly flat (Nawa, 2008). The former seems to be more easily detected during the screening process. Moreover, the quality of bowel cleaning determines the accuracy of colonoscopy screening (Parente et al., 2009). Therefore, poor bowel preparation often leads to loss of optimal visualization of proximal intestinal mucosa and thus misses diagnosis (Shroff, 2014).

For gender, this study indicated that the incidence of CRC fell by 27 % in males and 15 % in females after sigmoidoscopy screening (including colonoscopy). This may be due to the gender difference in the incidence of CRC. A report showed that from 1990 to 2019 the incidence of CRC was higher in males. The age-standardized incidence of CRC in males was 1.5 times higher than that in females (Global, regional, and national burden of colorectal cancer and its risk factors, 2019). The large incidence base made sigmoidoscopy screening more beneficial for males. In terms of mortality, there was a 32 % reduction in the males screening group and a 15 % reduction in females screening group compared to the control group. A study mentioned that due to its anatomical and physiological peculiarities, the transverse colon of females was longer than that of males, which might make females susceptible to incomplete screening, making it more difficult to detect tumors during screening (Saunders, 1996).

In summary, it was not difficult to see that the effectiveness of sigmoidoscopy screening (including colonoscopy) was related to the site of CRC and gender. Taking these two factors into account, this study demonstrated that compared to controls, sigmoidoscopy screening resulted in a 39 % reduction in the incidence of distal CRC in males, a 27 % reduction in the incidence of distal CRC in females, a 12 % reduction in the incidence of proximal CRC in males, and a 1 % reduction in the incidence of proximal CRC in females. In terms of mortality, comparing with the control group, the above trends could also be observed, i.e., a 48 %, 27 %, 14 %, and 8 % reduction in the mortality of males with distal CRC, females with distal CRC, males with proximal CRC, and females with proximal CRC, respectively. Notably, sigmoidoscopy screening appeared to have little impact in terms of incidence and mortality of proximal CRC in females. A systematic review pointed out that the proportion of right colon cancer (proximal colon cancer) was higher in females than in males (Hansen and Jess, 2012), and the right colon cancer was less differentiated and more advanced than left colon cancer (distal colon cancer) (Nawa, 2008, Benedix, 2010).

As for all-cause mortality, no statistical difference was found between the two groups in this study, either for males or females. A similar conclusion was reached in a network *meta*-analysis by Jodal *et al*. The reason for this may be that the participants involved in this study are mainly between the ages of 55 and 74. As a matter of common knowledge, chronic and degenerative diseases are the leading causes of death in the elderly population (Doll, 1995). Data from the 2019 Global Burden of Disease, Injury and Risk Factors Study (GBD 2019) demonstrated that ischemic cardiomyopathy, stroke, chronic obstructive pulmonary disease, Alzheimer's disease and other dementia, and lower respiratory tract infection were the top five causes of death in people aged over 70 (Global, regional, and national burden of diseases and injuries for adults 70 years and older, 2019). Besides, although the benefits of endoscopic screening have been fully well established and have become the primary means of CRC screening in the United States (Rex, 2015), low participation rate remains a common problem, especially for colonoscopy screening (Chen, 2019, Swan, 2010, Inadomi, 2012). For this study, the lowest screening rate in the intervention group was as low as 19.5 %. Among them, adverse reactions during endoscopic screening may be a major cause, such as bleeding, perforation, anesthesia complications, etc., which may be harmful to the population, especially the elderly (Wolf, 2018). Finally, all-cause mortality requires a long period of time to be tested, and the follow-up duration of the currently included RCTs may not be sufficient to meet this requirement. Based on these, it is speculated that CRC screening cannot reduce all-cause mortality when the mortality risks of other diseases are highly competitive, participation enthusiasm is low, the risk of complications from endoscopic screening is high, and follow-up time is inadequate. Therefore, the question of whether sigmoidoscopy screening (including colonoscopy) benefits the entire population seems worthy of consideration.

Eventually, the main strength of this study is that based on the latest RCTs, it once again systematically elucidated the relationship between sigmoidoscopy (including colonoscopy) and CRC incidence, CTC mortality and all-cause mortality. More importantly, this study also conducted subgroup analyses according to the site of CRC, gender and CRC in different sites of different genders, forming a more comprehensive understanding of the effectiveness of endoscopic screening. However, there are still some limitations. First, it is unfortunate that the screening methods included in the study were mainly sigmoidoscopy, and only one was colonoscopy screening. The limited scope of sigmoidoscopy screening might somewhat diminish the screening rate of proximal CRC and might not have adequately demonstrated the effectiveness of colonoscopy. Nevertheless, statistics indicated that for proximal CRC, both incidence and mortality were significantly lower than those in the non-screened group. Therefore, the role of sigmoidoscopy in the screening of CRC is positive. Second, due to the limited number of studies included, more diverse subgroup analyses were not performed, such as age stratification, comparison of the effectiveness between sigmoidoscopy and colonoscopy, etc. Third, the participants included in this study were mainly from areas with high prevalence of CRC, and these results may not be directly generalizable to countries with low prevalence of CRC.

## 5 Conclusion

This *meta*-analysis suggested that sigmoidoscopy screening (including colonoscopy) was effective in reducing the incidence and mortality of CRC in the general population, and was related to the site of CRC and the gender of participants. Meanwhile, large-scale, multi-center RCTs on endoscopic screening are expected to be conducted for further verification.

## 6 Authors’ contributions

All authors had read and approved the manuscript. Dongying Wang and Qian Xu writing manuscript; Senjie Dai performing procedures and data analysis; Yueming Zhang and Fulin Ding contribution to writing the manuscript; Linling Ji contribution to drafting conception and design.

## Funding source

This research did not receive any specific grant from funding agencies in the public, commercial or not for profit sectors.

## CRediT authorship contribution statement

**Dongying Wang:** Writing – original draft. **Qian Xu:** Writing – original draft. **Senjie Dai:** Formal analysis, Data curation. **Yueming Zhang:** Writing – original draft, Investigation. **Fulin Ding:** Writing – original draft, Investigation. **Linling Ji:** Supervision, Software, Formal analysis, Conceptualization.

## Declaration of competing interest

The authors declare that they have no known competing financial interests or personal relationships that could have appeared to influence the work reported in this paper.

## Data Availability

The datasets supporting this article’s conclusions are included within the article and its additional files.
