# Supplementary material for: Effects of sigmoidoscopy screening (including colonoscopy) on colorectal cancer: A meta-analysis based on randomized controlled trials
Source: Prev Med Rep. 2024 Feb 1;39:102636. doi: 10.1016/j.pmedr.2024.102636 (PMC10847765; doi:10.1016/j.pmedr.2024.102636)
Supplement: Supplementary data 1 [file mmc1.docx]

**Additional files**

Supplementary Figure 1. Summary of the risk of bias based on the evaluation domains listed in the Cochrane Collaboration Risk of Bias Tool.

Supplementary Figure 2. Proportion of risk of bias based on the evaluation domains listed in the Cochrane Collaboration Risk of Bias Tool.

Supplementary Figure 3. Sensitivity analysis of the relationship between sigmoidoscopy screening (including colonoscopy) and the incidence of CRC.

Supplementary Figure 4. Egger's test for the relationship between sigmoidoscopy screening (including colonoscopy) and the incidence of CRC (p=0.435).

Supplementary Figure 5. Sensitivity analysis of the relationship between sigmoidoscopy screening (including colonoscopy) and CRC mortality.

Supplementary Figure 6. Egger's test for the relationship between sigmoidoscopy screening (including colonoscopy) and CRC mortality (p=0.051).

Supplementary Figure 7. Sensitivity analysis of the relationship between sigmoidoscopy screening (including colonoscopy) and all-cause mortality.

Supplementary Figure 8. Egger's test for the relationship between sigmoidoscopy screening (including colonoscopy) and all-cause mortality (p=0.228).

Supplementary Table 1. Characteristics of included studies.
