# Supplementary material for: Effects of sigmoidoscopy screening (including colonoscopy) on colorectal cancer: A meta-analysis based on randomized controlled trials
Source: Prev Med Rep. 2024 Feb 1;39:102636. doi: 10.1016/j.pmedr.2024.102636 (PMC10847765; doi:10.1016/j.pmedr.2024.102636)
Supplement: Supplementary data 2 [file mmc2.docx]

**Supplementary Table 1.** **Characteristics of included studies.**

| Autor (Year) | Registration mark | Age | No. of participants | | Classification of CRC | Dropout rate | | Trial exclusion criterion | Outcomes | |
| --- | --- | --- | --- | --- | --- | --- | --- | --- | --- | --- |
|  |  |  | Male | Female |  | Intervention | Control |  | Primary | Secondary |
| Atkin  (2017) | ISRCTN  28352761 | 55-64 | 83334 | 86700 | Distal cancer: C18.7, C19, and C20;  Proximal cancer: C18.0~C18.6 | NR | | Participant had a history of CRC, adenomas, or inflammatory bowel disease; a life expectancy of less than 5 years; received a flexible sigmoidoscopy or colonoscopy within the previous 3 years; or were unable to provide informed consent | CRC incidence and mortality | Distal and proximal CRC incidence and mortality and all-cause and non-CRC mortality, and the number needed to screen to prevent one colorectal cancer diagnosis or death |
| Bretthauer  (2022) | NCT  00883792 | 55-64 | 42399 | 42186 | CRC:  C18~C20 | NR | | Participant had previously undergone screening and who not lived in one of the four countries where the trial was conducted | CRC incidence and mortality | All-cause mortality |
| Holme  (2018) | NTC  00119912 | 50-64 | 49127 | 49551 | NR | NR | | Participant had a history of CRC | CRC incidence and mortality | Distal and proximal CRC incidence and mortality and in men and women |
| Miller  (2019) | NCT  00002540 | 55-74 | 76678 | 78209 | NR | 13.0% | 17.1% | Participant had a history of prostate, lung, colorectal, or ovarian cancer and undergoing cancer treatment | Cause-specific mortality rates for each of the PLCO cancer sites^a^ | Overall and tumor stage incidence |
| Senore  (2022) | ISRCTN  27814061 | 55-64 | NR | | Distal cancer: C18.6 | 3.5% | 4.0% | Participant had died or been diagnosed with CRC before randomization | CRC incidence and mortality | Distal and proximal CRC incidence and mortality and all-cause mortality |

NR: Not reported; CRC: Colorectal cancer.

^a^ Prostate, lung, colorectal, and ovarian cancer.
