# Supplementary figures and images for: Effects of sigmoidoscopy screening (including colonoscopy) on colorectal cancer: A meta-analysis based on randomized controlled trials
Source: Prev Med Rep. 2024 Feb 1;39:102636. doi: 10.1016/j.pmedr.2024.102636 (PMC10847765; doi:10.1016/j.pmedr.2024.102636)

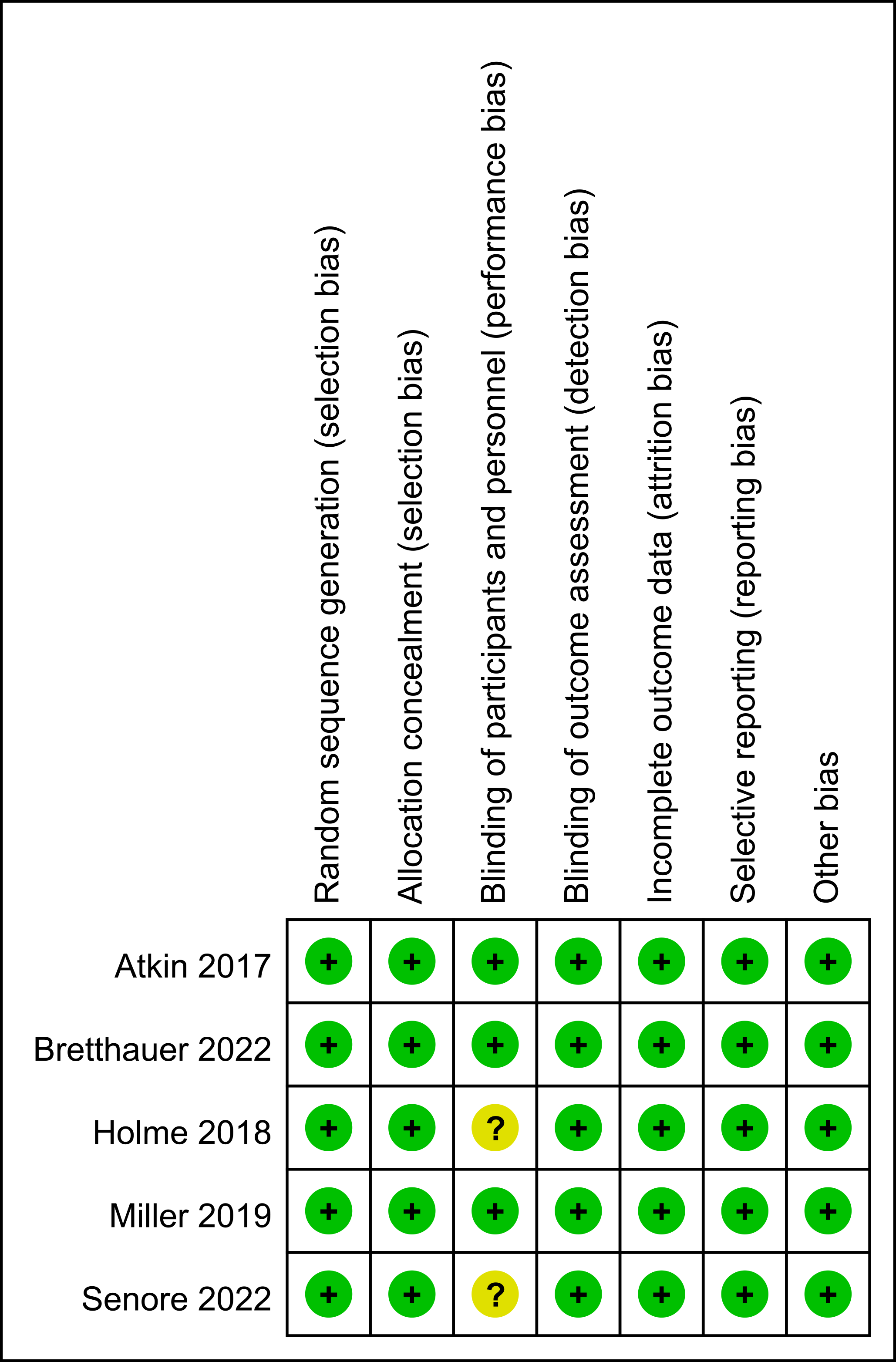

Supplement: Supplementary data 3 [file mmc3.zip › Supplementary Figure 1.tif]

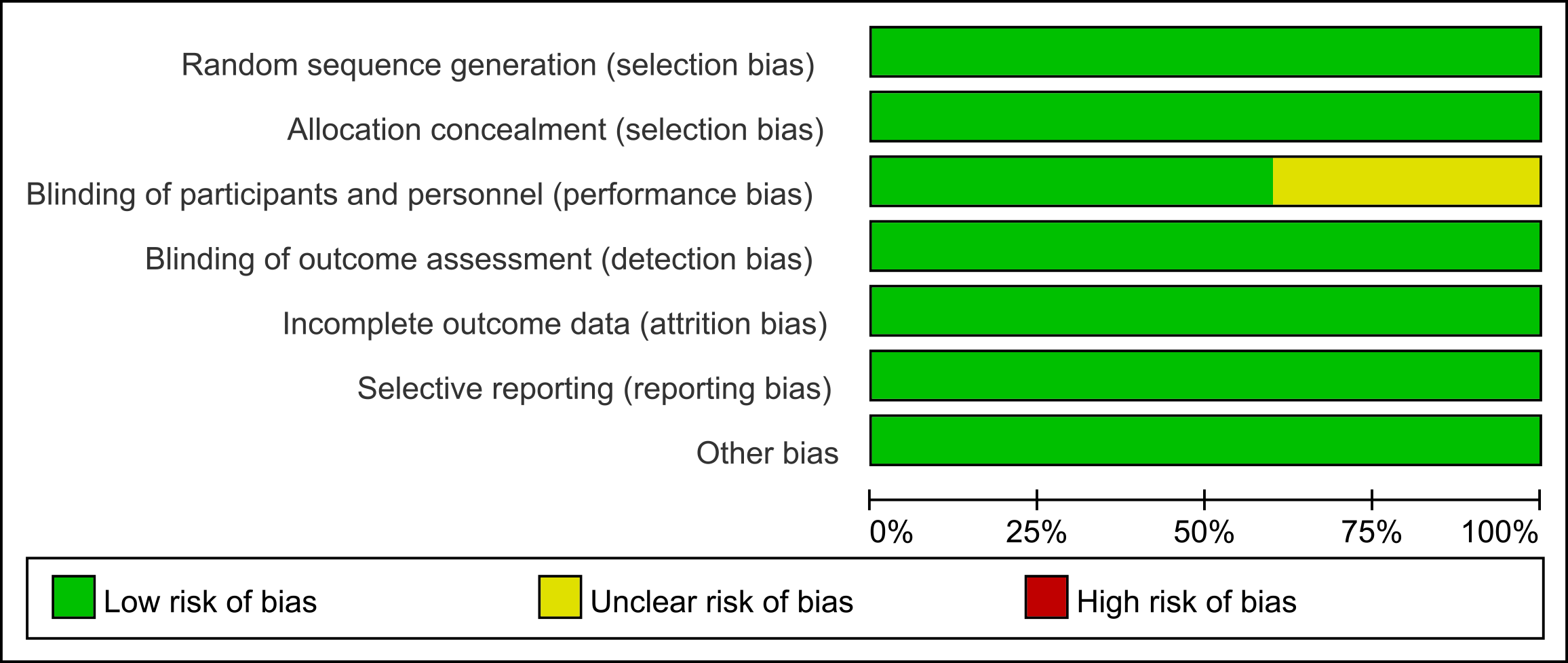

Supplement: Supplementary data 3 [file mmc3.zip › Supplementary Figure 2.tif]

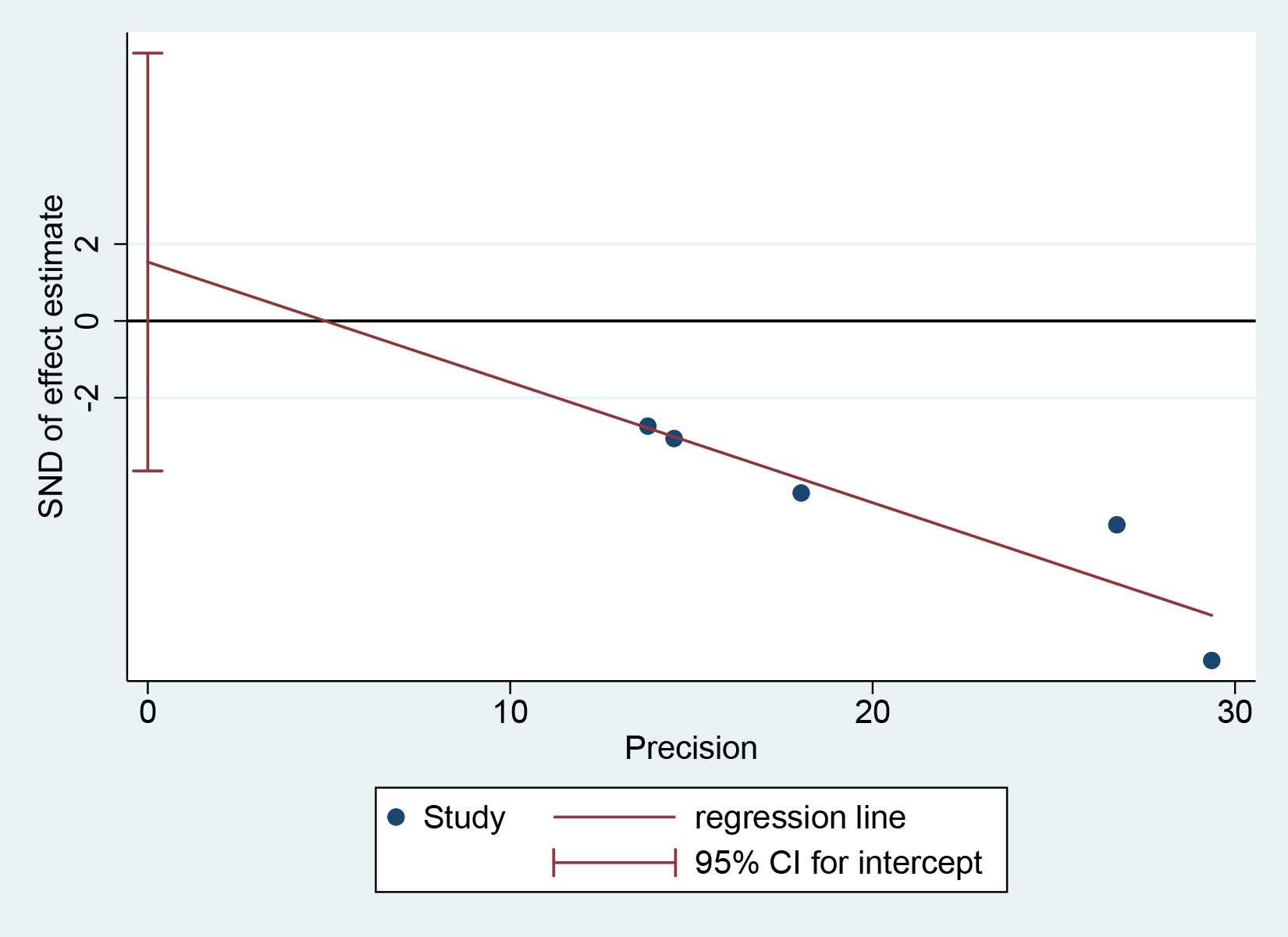

Supplement: Supplementary data 3 [file mmc3.zip › Supplementary Figure 4.tif]

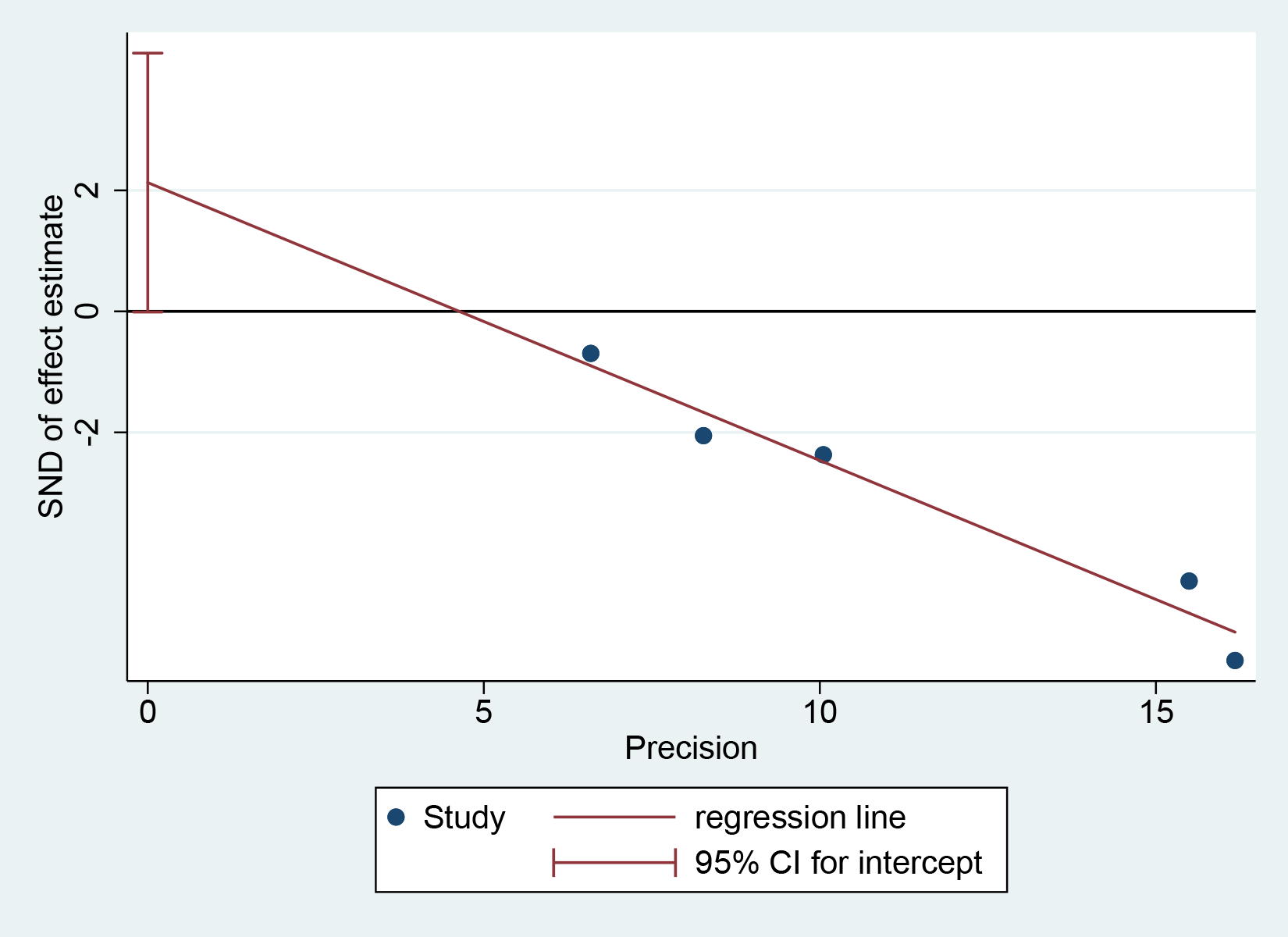

Supplement: Supplementary data 3 [file mmc3.zip › Supplementary Figure 6.tif]

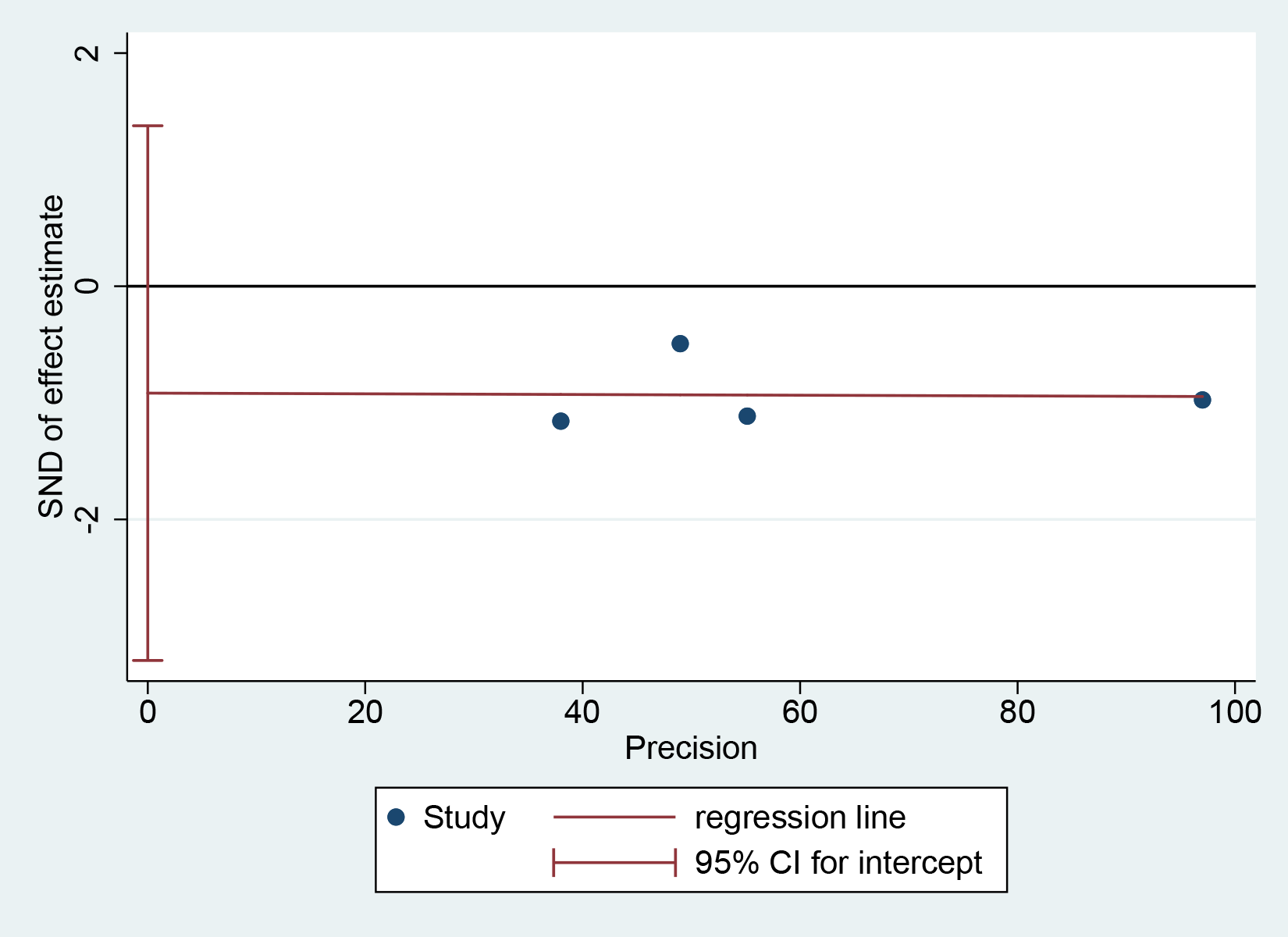

Supplement: Supplementary data 3 [file mmc3.zip › Supplementary Figure 8.tif]
